# Portion size: review and framework for interventions

**DOI:** 10.1186/1479-5868-6-58

**Published:** 2009-08-21

**Authors:** Ingrid HM Steenhuis, Willemijn M Vermeer

**Affiliations:** 1 VU University, Department of Health Sciences and EMGO+ Institute for Health and Care Research, De Boelelaan 1085, 1081 HV Amsterdam, the Netherlands

## Abstract

The prevalence of overweight and obesity has increased. A strong environmental factor contributing to the obesity epidemic is food portion size. This review of studies into the effects of portion size on energy intake shows that increased food portion sizes lead to increased energy intake levels. Important mechanisms explaining why larger portions are attractive and lead to higher intake levels are value for money and portion distortion. This review also shows that few intervention studies aiming to reverse the negative influence of portion size have been conducted thus far, and the ones that have been conducted show mixed effects. More intervention studies targeted at portion size are urgently needed. Opportunities for further interventions are identified and a framework for portion size interventions is proposed. Opportunities for intervention include those targeted at the individual as well as those targeted at the physical, economic, political and socio-cultural environment.

## Introduction

Overweight and obesity are increasing problems in western societies. Environmental factors contribute to the obesity epidemic [1] by promoting energy intake and limiting opportunities for energy expenditure [2]. A strong environmental factor influencing energy intake is food portion size [3-6]. Although research on the actual development of portion sizes is limited, it is clear that portion sizes have increased over the past decades [7-11]. Studies have been conducted in the United States [7-10] and in Denmark [11]. These studies show that, since the 1970s, portion sizes of especially high energy-dense foods, eaten inside as well as outside the home, have increased. This accounts for both amorphous foods and foods served in units [9]. Fast-food restaurants, for example, have shown a trend over the last decades to supersize their portions, and have introduced large and mega meals [9-11]. Another example is the increased package sizes of products sold in supermarkets, such as sugar-sweetened beverages [11].

Food portions in the United States tend to be larger than in Europe. However, in Europe, portion sizes have also increased [5,10,11]. Increased portion sizes may lead to increased energy intake levels. Studies on interventions that aim to reverse this trend are scarce, and urgently needed. In this article, firstly we review the effects of portion size on energy intake, followed by possible explanations for this relationship. Next, we assess the currently available interventions and their effectiveness. To conclude, we identify further opportunities for interventions aimed at portion size and propose a framework for portion size interventions.

## Methods

### Search strategy

For this review, firstly we asked: What is the effect of portion size on energy intake? Secondly, we assessed the effects of currently available portion size interventions on food intake. Studies were identified using the PUBMED database, the Cochrane Library and the Web of Science (ISI). The following keywords were used for the first question: 'portion size'; 'energy intake'; 'food intake'; and 'food consumption'. For the second part, the keywords 'portion size'; 'intervention'; or 'programme' were used. Furthermore, studies were also identified based on references of the found articles.

### Inclusion and exclusion criteria

Only studies with adults as research population were included in this review. However, intervention studies with a mixed study sample, but consisting mainly of adults, were also included. Studies with less than 20 subjects were excluded in all cases. Food intake had to be an outcome measure of the study to be included in the review. More specifically, food intake of the product whose portion size was manipulated had to be an outcome measure (for example, instead of only the food intake of a non-manipulated subsequent meal). In addition, for the second part, intervention studies that used food selection as an outcome measure were also included.

For the first question, studies varying only the package format and not the actual portion size were excluded (for example 30 grams in a small package versus 30 grams in a large package). Regarding study design, between subjects designs were included as well as within subjects cross-over designs for the first research question. Since studies into the effectiveness of interventions aimed at portion size are very scarce, no further requirements were defined regarding research design for the second question.

## Portion size and energy intake

Do increased portion sizes lead to increased energy intake levels? Thirteen studies met the inclusion criteria and investigated this relationship, mostly using a within subject's cross-over design (see Table 1) [12-24]. The larger portion sizes used in the studies varied from 125% of the control portion to up to 500% the control portion, but most studies investigated portion sizes between the control size and twice the control size. All studies showed that people's energy intake increases when offered a larger portion. This also accounts for food with an unfavourable perceived taste, i.e. stale popcorn [23].

**Table 1:** Studies into the effects of portion size on food intake

**First author, year (reference)**	**Study design**	**Respondents**	**Type of food**	**Main outcome**
Diliberti,2004 [12]	Between subjects, parallel group design, with two different portion sizes (100%^a^, 152%)	Cafeteria visitors at a university campus, n = 180	Baked pasta in cheese sauce (54% fat, energy density 1.7 kcal/g)	-Increased energy intake when served a larger portion, 43% more (172 kcal) -Overall extra energy intake of entire meal 25% (172 kcal)

Fisher, 2007 [13]	Within subjects cross over design, with two different portion sizes (100%^a^, 200%)	Low income Hispanic and African American mothers, n = 58	Macaroni & cheese (1.51 kcal/g), apple juice (0.47 kcal/g), crackers (4.62 kcal.g), chicken (1.73–2.42 kcal/g), rice (0.8 kcal/g), cereal (4.0 kcal/g)	-Increased energy intake when served a larger portion, 21% more (270 kcal), over 24 hour period

Flood, 2006 [14]	Within subjects cross over design, with two different portion sizes (100%^a^, 150%) (and three different beverages)	Adults, n = 33 (aged 18–45)	Beverages (regular cola (0.4 kcal/g), diet cola, water)	-Increased beverage intake when served a larger portion, 10% more for women, 26% for men (regular cola)

Kral, 2004 [15]	Within subjects cross over design, with three different portion sizes (100%^a^, 140%, 180%) (and three different energy densities)	Women (aged 20–45), n = 39	Italian pasta bake (25% fat, 60% carbohydrate, 15% protein, 1.25 kcal/g–1.75 kcal/g)	-Increased food intake when served a larger portion, 20% more food intake when served the largest portion compared to the smallest portion -Combined effect with energy density: 56% more energy intake when served the largest higher energy dense portion compared to the smallest lower energy dense portion (225 kcal)

Raynor, 2007 [16]	Random 2(small amount or large amount, 100%^a^, 200%) × 2 (small unit or large unit) between subjects design	Adults (aged 18–30), n = 28	Potato chips, cheese crackers, cookies, candy	-Increased energy intake when served a larger portion, 81% (2246 kcal), over three day period -No effect of package unit size

Rolls, 2002 [17]	Within subjects cross over design, with four different portion sizes (100%^a^,125%, 150%, 200%)	Adults (aged 21–40), n = 51	Macaroni & cheese (1.63 kcal/g)	-Increased energy intake when served a larger portion (resp. 12% more (64 kcal), 19% more (105 kcal), and 30% more (161 kcal)

Rolls, 2004 [18]	Within subjects cross over design, with five different portion sizes (100%^a^, 150%, 204%, 357%, 507%)	Adults (aged 20–45), n = 60	Potato chips (5.4 kcal/g)	-Increased energy intake when served a larger portion, 184 kcal more for women when comparing largest vs smallest portion, for men 311 kcal -No short term compensation at dinner

Rolls, 2004 [19]	Within subjects cross over design, with four different portion sizes (100%^a^, 134%, 167%, 200%)	Adults (aged 20–45), n = 75	Deli-style sandwich, (2.4 kcal/g)	-Increased energy intake when served a larger portion, 31% more for women when comparing largest vs smallest portion(159 kcal), for men 56% more (355 kcal)

Rolls, 2006 [20]	Within subjects cross over design, with three different portion sizes (100%^a^, 150%, 200%)	Adults (aged 19–45), n = 32	Complete daily menu (varying from 0.2 kcal/g (vegetable side dish) to 5.5 kcal/g (snack foods)	-Increased energy intake when served larger portions, for all food categories, resp. 16% more (women 335 kcal/day, men 504 kcal/day) and 26% more (women 530 kcal/day, men 812 kcal/day) -No compensation over two day time period

Rolls, 2007 [21]	Within subjects cross over design, with two different portion sizes (100%^a^, 150%)	Adults (aged 20–40), n = 23	Complete daily menu, each day different	-Increased energy intake when served larger portions, for all food categories except fruit as afternoon snack and vegetables, average increase in energy intake 423 kcal/day -No compensation over 11-day time period

Wansink, 2001 [22]	2 (medium or large container, 100%^a^, 200%) × 2(perceived favourable vs unfavourable taste) between subjects design	Moviegoers (aged 11–89), n = 151	Popcorn	-Increased food intake when served a larger portion, for both perceived favourable and unfavourable taste, 53% more

Wansink, 2005 [23]	Random 2 (medium or large container, 100%^a^, 200%, × 2 (fresh or stale) between subjects design	Adult moviegoers, n = 158	Fresh and stale popcorn	-Increased food intake when served a larger portion, for both fresh and stale popcorn, resp. 45% and 34%

Wansink, 2005 [24]	Random between subjects, parallel group design (normal bowl vs self refilling bowl)	Adults (ages 18–46), n =	Soup	-Increased energy intake when served a larger portion without accurate visual cue, 73% more (113 kcal)

^a ^smallest portion is referred to as 100%

Effects of at least 30% higher consumption levels due to portion size are reported frequently [12,16,17,19,22-24], with larger effects for larger portion sizes (see Table 1). The effects have been shown for a variety of foods, such as macaroni [17], a pre-packaged snack [18], beverages [14] or popcorn offered in a cinema setting [22]. Larger effects are found for men [18-20], compared to women. If the increase in portion size was combined with a higher energy density, even larger effects on energy intake were observed [15]. Furthermore, research showed that the effects of portion size can persist over several days, with no indication of meal-to-meal compensation [20,21]. Rolls et al [21] showed that the average increase in caloric intake owing to 50% larger portions did not decline over a period of eleven days, and resulted in a cumulative increase of, on average, more than 4600 kcal during the eleven-day research period.

### Explanations: Portion distortion and value for money

Important factors found in the literature, explaining why people buy and eat larger portion sizes than they actually need, are the notions of 'value for money' and 'portion distortion'. Larger portions are made attractive by offering more value for money, i.e. having a lower price per unit. Lower unit costs also explain why larger package or portion sizes lead to a higher user volume [6,25,26]. Based on qualitative focus group interviews, it seems that consumers experience the lower price per unit in case of larger portions as a natural pattern and are used to it (March 2007; unpublished data).

Next, continuous exposure to larger food portion sizes contributes to 'portion distortion' among consumers [11,27]. People experiencing portion distortion perceive larger portion sizes as an appropriate amount to consume at a single occasion [28]. It also refers to the fact that individuals do not realize that their portion size commonly exceeds the serving size [29]. With respect to portion distortion, a number of aspects are relevant. First, larger portions have become standard and, as a consequence, consumers have difficulty selecting amounts of food that are appropriate for their weight and activity levels [30]. Second, market place portions differ increasingly from recommended standard portion sizes defined by federal agencies [30]. In fact, market place portions are often three to four times larger than the recommended portion size, while consumers perceive market place portions as standard portions [31].

Several studies have shown that people tend to select substantially larger portions than the recommended portion sizes [28,29,32,33]. Third, labels on food packaging are not always clear with respect to the serving size. Sometimes, unrealistic small serving sizes are used on food packages in order to give consumers a positive impression about the number of servings in one package and the caloric content [29]. Similarly, using the terms 'small', 'medium' and 'large' also creates confusion, as people's interpretation of these terms differs [34]. A fourth factor relevant in portion distortion is the 'unit bias' people might experience. Geier et al [35] define unit bias as 'a sense that a single entity is the appropriate amount to engage, consume or consider'. The size of the unit or package sets a consumption norm for consumers, which might not be an appropriate norm in accordance with food recommendations [35,36]. Many people interpret package size as a single serving size and are unaware of the fact that a package contains multiple servings [37]. Fifth, and finally, tableware might also contribute to portion distortion, although study results are inconclusive as yet. The vertical-horizontal illusion is well known: people only use the vertical dimension to estimate portion size [38]. Also, it seems that people serve themselves more food if using a larger bowl [39,40]. However, for plate sizes, this finding could not be replicated [41].

Once larger portions have been selected because of the value for money and portion distortion principle, passive over-consumption is likely to occur. In particular, people tend to overeat palatable, high energy-dense (e.g. high in fat) foods, without deliberate intention [42].

## Interventions

### Intervention studies

Is it possible to reverse the trend towards larger portions and the consequently higher energy intake levels? Despite the fact that a broad range of interventions aimed at portion size have been suggested in the literature, very few intervention studies aimed at portion size have been conducted thus far. Only five studies were found that met the inclusion criteria for the intervention studies (see Table 2) [43-47]. It must be noted that two of these intervention studies were conducted among relatively small samples [46,47], with 24 and 33 respondents respectively. Also, three of these five studies were conducted among a relatively young and healthy population; i.e. college students [43,45,46]. Few other studies describing an intervention aimed at portion size and targeted at adults were found. However, these studies did not have food intake or selection as their outcome measurement, or were not evaluated at all [27,48-52]. These interventions were developed to target the improvement of consumers' portion size estimation skills, and to educate people about appropriate portion sizes [27,48-52]. Of the five included studies, two were targeted at reducing portion sizes [45,46].

**Table 2:** Studies into the effectiveness of interventions aimed at portion size

**First author, year (reference)**	**Intervention**	**Study design**	**Respondents**	**Effects**
Antonuk, 2006 [43]	Package nutritional information; dual column labelling: not only nutritional information for one serving but also for the entire package	Random between subjects, parallel group design (nutritional information about serving size ('single column') vs nutritional information about serving size *and *entire package ('dual column')	College students, n = 112	-Non dieters ate significant less of a snack food when confronted with dual labelling -No effect on intake of dieters

Harnack, 2008 [44]	Elimination of value size pricing and calorie labelling of different fast food portion sizes	Random 2(value pricing or normal pricing) × 2(calorie labelling or no labelling) between subjects design	Regular fast food restaurant visitors, adolescents and adults, n = 594	-No differences in energy composition of ordered meals

Lieux, 1992 [45]	Maximum of 1 hot entrée per person, no larger portions on request	Observational within subjects study, with four measurements	College students, n = 214	- Men increased selection of other foods so that energy intake remained the same - Women chose the same foods

Rolls, 2006 [46]	25% Reduction in portion size and 25% reduction in energy density (i.e. by substituting full-fat ingredients by low-fat ingredients or by increasing the proportion of fruit & vegetables	Within subjects cross over design with four conditions	Young women, n = 24	-Independent effects of reducing portion size and energy density on energy intake found, effects sustained over 2 days -Stronger effect of reduction in energy density -No effect on ratings of hunger and fullness

Ueland, 2009 [47]	Portion size information; written descriptions with a comparison to a reference amount, i.e. " this is 1,5 times a normal portion of this pasta"	Within subjects crossover design	Normal weight adults, n = 33	-No effect on on total food intake -No effect on satiety

A portion size reduction of 25%, studied in a laboratory setting, was effective in decreasing energy intake. Moreover, reducing the energy density, while keeping the same portion size, led to a larger decrease in energy intake [46]. However, a study into reducing portion sizes in a college setting showed no effects on total energy intake [45]. Lieux and Manning [45] studied an intervention based on limiting portion sizes of hot entrées in a dining facility at an American university. Portion size labelling or portion size information seems to be ineffective in decreasing energy intake [44,47]. In Harnack's study [44], portion size labelling was combined with another pricing structure (i.e. standardized pricing: the same price per gram for small and large portions; instead of value size pricing: the per unit cost decreases as portion size increases), which was also ineffective in changing the caloric intake. As customers are not used to standardized pricing, it might be that repeated exposure is necessary in order to achieve an effect [44].

### Suggestions for individual and environmental interventions aimed at portion size

Several suggestions for interventions aimed at portion size have been given in the literature (see Table 3). Interventions can be targeted at the individual, comprising the education of consumers. Educational interventions should address awareness and teach behavioural strategies for portion control at home as well as, for example, in restaurants [4,7,10,11,28,29,31,53,54] (see Table 1 for more details regarding the suggested interventions).

**Table 3:** Opportunities for interventions aimed at portion size

**Type of intervention**	**Suggested intervention**	**First author, year (reference)**
*Individual* [Purple]	*Increase awareness *among consumers - explain that the amount eaten is important, not only what is eaten - explain the relationship between portion size, calories and obesity - explain the relation between product packaging, media and eating habits	e.g. Nielsen, 2003 [7]; Young, 2007 [10]; Mathiessen, 2003 [11]; Rolls, 2003 [53]; Burger, 2007 [32]; Schwartz, 2006 [28]

	Education about *serving sizes and portion sizes*	Hogbin, 1999 [31]

	Educate people to eat *satisfying portions of low energy dense foods *instead of restricting all portions	Ello-Martin, 2005 [54]

	Educate people on strategies regarding *controlling portion size in a restaurant setting*, for example - ordering reduced-size portions - saving part of entree for another meal - sharing	Ledikwe, 2005 [4]

	Educate people on strategies regarding *controlling portion size at home*: - repackage foods into smaller units - plate smaller dinner portions in advance - transfer food to a plate or bowl instead of eating from the package - reduce the visibility of stockpiled foods - reduce the convenience of stockpiled food (f.e. by freezing them) - use tall, narrow glasses - use smaller bowls and plates - use smaller spoons when serving oneself or when eating from a bowl	Wansink, 2004 [36]

*Environmental*		

Physical environment [Blue]	*Decrease portion sizes *served in restaurants and of packaged food in grocery stores incrementally, without knowledge of consumers	Ayala, 2006 [48]; Condrasky, 2007 [33]

	Serve *larger portions *of *healthy*, low energy-dense foods	Wansink, 2005 [24]

	*Reduce energy density *of food products/meals, keeping satisfying portion size	Ledikwe, 2005 [4]; Rolls, 2003 [53]

	Provide a *wider range of portion sizes*	Ello-Martin, 2005 [54]; Ledikwe, 2005 [4]; Osterholt, 2006 [56]; Rolls, 2003 [53]

	Use *commercially packaged meals *that are *controlled for portion size *and energy density	Ello-Martin, 2005 [54]

	*Food labels *with useful and clear information about portion size	Bryant, 2005 [29]; Rolls, 2003 [53]; Schwartz, 2006 [28]

	*Point-of purchase information *about portion size (restaurants)	Rolls, 2003 [53]; Young, 2007 [10]

Economic environment [Yellow]	Attractive *pricing strategies *to promote smaller portions - same value for money for small and large portions - discount on reasonable sized portions	Ello-Martin, 2005 [54]; French, 2005 [25]; Ledikwe, 2005 [4]

	*Incentives for industry *to reduce portion sizes	Rolls, 2003 [53]

Political environment [Green]	*Portion size requirements *on all foods in a la carte lines at schools	Hartstein, 2008 [58]

	More consistent and *realistic serving size standards *that are achievable	Young, 1995 [57]

Socio-cultural environment [Red]	*Improve nutrition knowledge of chefs' *of restaurants, about calories, portion size, energy density	Condrasky, 2007 [33]

Further, environmental interventions are important as well, since increased portion sizes are part of a changed food environment. Physical, economic, political and socio-cultural aspects of the environment can be distinguished according to the ANGELO-grid (ANalysis Grid for Environments Linked to Obesity) [1]. The physical environment refers to available options to make a healthy choice; the economic environment refers to the cost of healthy choices; the political environment refers to rules and regulations that may influence healthy choices; and the socio-cultural environment refers to social and cultural norms influencing healthy choices [1,55]. Interventions aimed at portion size can be put in place in all four types of environment (see Table 1). Decreasing portion sizes, or a wider range of available portion sizes, are examples of interventions aimed at the physical environment [10,23,28,29,33,53,54,56,57]. Pricing strategies to make smaller portions more attractive aim to alter the economic environment [4,26,53,54]. Possible interventions in the political environment are portion size requirements in certain settings, such as schools, or formulating realistic serving size standards [33,48,53,58]. Finally, interventions in the socio-cultural environment could be directed at chefs in restaurants, aimed at changing their knowledge, attitudes and skills regarding portion size and thereby influencing their customers' food consumption (see Table 3 for more details regarding the suggested interventions).

### Portion size intervention framework

**Figure 1:**
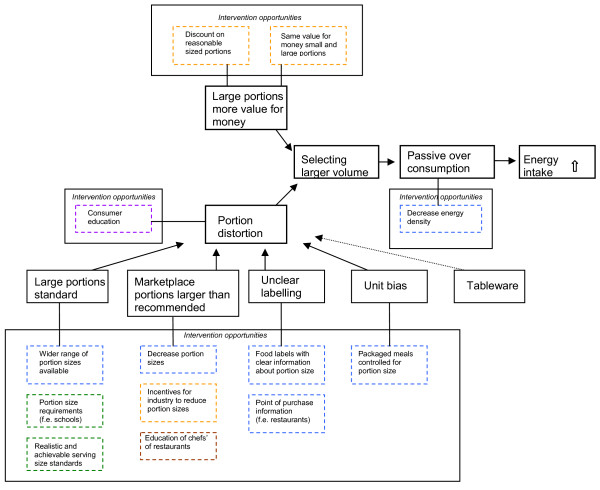
Framework for portion size interventions

The suggested interventions target different aspects of portion size, which contribute to a higher energy intake mentioned before (see 'Portion distortion and value for money'). Figure 1 shows a framework for portion size interventions. The underlying factors causing portion distortion can be diminished by means of environmental interventions, mainly in the physical and political environment. Alongside this, education of consumers may help them to cope with an environment filled with portion distortive factors. Furthermore, proposed pricing strategies direct the value for money principle. Most of the suggested interventions are targeted at the selection of food, which is of great value because once a larger portion is selected, over-consumption is very likely to occur. Yet, by decreasing the energy density of food products and meals, one is still able to select a larger volume, while having fewer consequences for energy intake. Since value for money, as well as portion distortion phenomena, are less important using this strategy, this might be a promising alternative and attractive to both consumers and retailers.

### Feasibility

The feasibility of interventions targeted at portion size depends on the willingness of both consumers and point-of-purchase settings to accept these interventions. A qualitative study into consumer attitudes about portion size interventions indicated that consumers had particularly favourable attitudes towards a larger variety of portion sizes and pricing strategies, followed by labelling interventions (March 2007; unpublished data). Another qualitative study using semi-structured individual interviews with representatives of point-of-purchase settings, showed that most interventions aimed at portion size can be considered as innovative. Nonetheless, offering a larger variety of portion sizes and portion-size labelling were perceived as especially feasible interventions [59]. Also, O'Dougherty et al [60] showed that a third of fast-food restaurant patrons favoured a law requiring restaurants to change their pricing strategies and offer lower prices for smaller portions, instead of more value for money for larger portions.

## Conclusion

Portion sizes seem to have increased considerably over the last few decades. It is important to continue studying trends in actual portion size development, since not many studies are currently available. The same applies to studies into the long-term effects of increased portion sizes. This review summarizes the available evidence, demonstrating that increased portion sizes lead to increased energy intake levels. Important factors explaining why larger portions are attractive, and why they lead to higher intake levels, are related to value for money and portion distortion. Only few intervention studies have been conducted to target portion size. Interventions that have been tested were directed mainly towards the physical environment, namely portion size reduction and portion size labelling or information. So far, interventions have shown mixed effects. Intervention studies are urgently needed, to find out what type of interventions, targeted at portion size, are effective, in what setting, and among which target groups. These studies should focus on educational programmes, but also on interventions in the physical, economic, politic and socio-cultural environments.

## Competing interests

The authors declare that they have no competing interests.

## Authors' contributions

IS conceived of the study, carried out the literature review and drafted the manuscript. WV participated in the literature review and helped to draft the manuscript. All authors read and approved the final manuscript.
